# The lonely glutamine tree in the middle of the infinite critically ill forest

**DOI:** 10.1186/s13054-021-03743-x

**Published:** 2021-09-17

**Authors:** Panagiotis Briassoulis, Stavroula Ilia, Efrossini Briassouli, Marianna Miliaraki, George Briassoulis

**Affiliations:** 1 Pediatric Intensive Care Unit, Postgraduate Program “Emergency and Intensive Care in Children Adolescents and Young Adults”, School of Medicine, University of Crete, Section 6D (delta), Office 03, Voutes, 71003 Heraklion, Crete Greece; 2 First Department of Internal Medicine - Propaedeutic, National and Kapodistrian University of Athens, Athens, Greece

**Dear Editor,**

We read with great interest the article by Dr. Smedberg et al. addressing the issue of plasma glutamine status at intensive care unit (ICU) admission [1]. As indicated in their paper, hyperglutaminemia (≥ 930 μmol/L) at admission is an independent mortality predictor. There are issues that we want to highlight and comment on.

First, in this cohort of patients, 85% had liver disease at admission. The study lacks a non-liver failure hyperglutaminemic and/or a liver-failure non-hyperglutaminemic control group to examine metabolic pathways, amino acid kinetics, and compared outcomes. In a recent study in critically ill patients enrolled from three ICUs in South Africa, 14.2% had high (median 898.9 µmol/L) plasma glutamine levels. Patients with a diagnosis of liver failure had the highest glutamine levels. In this study, mortality was higher in the low (< 420 μmol/L) compared to high (> 420 μmol/L) glutamine level (62.2% vs. 37.8%) [2]. We have previously demonstrated that high doses of L-alanine-glutamine or L-glutamine did not induce any of the T helper (Th)1, Th2, and Th17 cytokines in either healthy or septic human PBMCs at 4 and 24 h [3].

Second, the main finding of the study is that hyperglutaminemia at ICU admission was associated with a more than twofold higher mortality rate at six months (46%) compared to patients with normal or low plasma glutamine concentrations at admission (18%). But the high plasma glutamine concentration could have led to limit protein/amino acid intake in these patients. Why should an initial high glutamine level be associated with mortality at 6 months? The acute phase of critical illness is associated with variable patterns of plasma amino acid changes, characterized by decreasing, increasing trends, or no changes in plasma levels compared with the values found in healthy subjects [4]. As the disease progresses, the levels of different amino acids gradually decrease, increase, or fluctuate over time. We have previously shown that pediatric patients with septic shock (hospital mortality 22%) had lower levels of glutamine on ICU day 3 (410 vs. 726 μmol/L) and 5 (426 vs. 691 μmol/L), compared to patients with non-infectious critical illness (mortality 4%) [4].

Similar to other nutritional constituents, temporarily adapted to the acute phase of critical illness [5], hyperglutaminemia could have only been an epiphenomenon, obviously affected by acute liver damage at admission. Future explorations are thus encouraged to clarify the mechanisms underlying the metabolism of glutamine in critical illness-related organ failures.

## Data Availability

Not applicable.
