# Supplementary material for: The lonely glutamine tree in the middle of the infinite critically ill forest
Source: Crit Care. 2021 Sep 17;25:342. doi: 10.1186/s13054-021-03743-x (PMC8449425; doi:10.1186/s13054-021-03743-x)
Supplement: Related texts

# Reply from the authors

**Authors:** Marie Smedberg, Inga Tjäder, Olav Rooyackers, Jan Wernerman

**Affiliations:** grid.24381.3c 0000 0000 9241 5705 Department of Anesthesiology and Intensive Care, CLINTEC, Karolinska Institute and Perioperative Medicine and Intensive Care, Karolinska University Hospital Huddinge, Stockholm, Sweden

We thank Drs Briassoulis et al. for their interest in and comments on our recent publication [1]. As stated, the glutamine tree is indeed lonely in the infinite critically ill forest. The main reason for us to publish this piece of information was the lack of published materials over hyperglutaminemia in the adult critically ill population. Our first observation of admission hyperglutaminemia as a risk factor for mortality contained only 8 subjects [6]. The present study contains 26 subjects, or 19 subjects if the postoperative liver transplant patients with zero mortality are excluded. The small number of subjects makes statistical comparisons hazardous. In both these studies, we defined hyperglutaminemia for the critically ill as the cutoff (maximum of sensitivity + specificity) for 6-month mortality in a ROC curve including all study patients with an admission plasma glutamine value above the normal range for healthy subjects, which turned out to be identical (930 umol/L) in the two studies. The study by Blaauw et al. has a different design and contains a different patient cohort, but basically it confirms our results for the hyperglutaminemic subjects, although they use the upper normal level for healthy individuals to define hyperglutaminemia [2]. We agree with Drs Briassoulis et al. that prospective studies over the underlying mechanism behind the statistical connection between glutamine plasma concentrations and mortality outcomes in the critically ill are a future challenge for those of us that are interested in the “glutamine tree”.
